# Maternal Gestational Diabetes Influences DNA Methylation in the Serotonin System in the Human Placenta

**DOI:** 10.3390/life12111869

**Published:** 2022-11-13

**Authors:** Jae Yen Song, Kyung Eun Lee, Eun Jeong Byeon, Jieun Choi, Sa Jin Kim, Jae Eun Shin

**Affiliations:** Department of Obstetrics and Gynecology, College of Medicine, The Catholic University of Korea, Seoul 06591, Korea

**Keywords:** epigenetics, gestational diabetes, placenta, DNA methylation, serotonin

## Abstract

The offspring of mothers with gestational diabetes mellitus (GDM) are at a higher risk for metabolic dysregulation and neurodevelopmental impairment. Evidence suggests that serotonin, which is present in both the placenta and the brain, programs the development and growth of the fetal brain. In the current study, we tested the hypothesis that GDM affects the methylation of the serotonin transporter gene (*SLC6A4*) and serotonin receptor gene (*HTR2A*) in the placenta. Ninety pregnant women were included in this study. Thirty mothers were diagnosed with GDM, and sixty mothers served as controls in a 1:2 ratio. Ten CpG sites within the promoter regions of *SLC6A4* and *HTR2A* were analyzed using pyrosequencing. The relative expression of genes involved in DNA methylation was evaluated using real-time PCR. The average DNA methylation of placental *SLC6A4* was higher in the GDM group than in the control group (2.29 vs. 1.16%, *p* < 0.001). However, the average DNA methylation level of *HTR2A* did not differ between the two groups. *SLC6A4* methylation showed a positive correlation with maternal plasma glucose level and neonatal birth weight percentile and a negative correlation with the neonatal head circumference percentile. This finding suggests that epigenetic modification of the placental serotonin system may affect placental adaptation to a harmful maternal environment, thereby influencing the long-term outcome in the offspring.

## 1. Introduction

Maternal gestational diabetes mellitus (GDM) is one of the most prevalent adverse environmental stressors affecting the fetus. In South Korea, approximately 12% of infants are exposed to maternal GDM during pregnancy [1]. Numerous studies link maternal hyperglycemia to long-term metabolic and neurodevelopmental outcomes in offspring. Offspring of maternal GDM have an increased risk of developing non-communicable diseases, including diabetes, obesity, and cardiovascular disease [2,3]. They also show altered neurological development, such as lower cognition, education attainment, and autism spectrum disorder (ASD) [2,4,5]. Despite the relatively consistent associations found in previous studies, the underlying pathways and mediators linking maternal metabolic status to offspring outcomes remain unclear. Prenatal adversity may affect offspring outcomes through fetal programming [6]. DNA methylation, which affects gene expression across generations without changing the nucleotide sequence, has been widely explored and may serve as a link between maternal hyperglycemia and the neurodevelopment and overgrowth of offspring [7,8,9]. Using candidate genes in the serotonin system, we aimed to explore the DNA methylation in the placenta as a potential mechanism connecting an adverse prenatal environment and neonatal development.

Serotonin is a multifunctional neurotransmitter that regulates energy balance or mood, and its role in the pathogenesis of ASD, depression, and other psychopathological conditions [10,11] is well established. Serotonergic pathology has been implicated in metabolic syndrome and obesity [10]. Serotonin in the placenta influences fetal growth and neurodevelopment [12,13]. The human brain and placenta express *SLC6A4*, which encodes the serotonin transporter that plays a key role in regulating the serotonin system; a growing body of evidence has shown its association with obesity and metabolic diseases [14,15]. In addition, the 5-hydroxytryptamine receptor 2A gene (*HTR2A*), which encodes a serotonin receptor subtypes, has been identified in the placental trophoblast and fetal capillary endometrium and contributes to the pathogenesis of obesity and psychopathological conditions [16,17,18].

Previous research has primarily focused on associations between serotonin system abnormalities and infant neurobehavior; little is known about alterations in the placental serotonin system in GDM mothers or any causal effect on offspring outcomes. Therefore, this study aimed to compare DNA methylation levels in the serotonin systems in the fetal side of the placenta between GDM and control groups to understand placental adaptation to GDM. The secondary objective was to evaluate the relationship between DNA methylation in the placental serotonin system and maternal and neonatal outcomes.

## 2. Materials and Methods

### 2.1. Study Population

This study included placentas from GDM-complicated pregnancies (*n* = 30) and healthy control pregnancies (*n* = 60). Electronic medical records (EMRs) were used to identify eligible women with GDM and healthy women who underwent prenatal examination and cesarean delivery at Bucheon St. Mary’s Hospital between 2017 and 2021. At the time of sample collection, all patients provided written informed consent for the possible use of their samples in future studies. This study was approved by the Ethics Committee of the Clinical Research Coordinating Center of the Catholic Medical Center (HC19SNSI0088).

Women over 18 years of age and who delivered singletons were included in the study. Women with the following diagnoses were excluded from the study: pre-existing maternal renal disease, diabetes mellitus, hypertension, fetal congenital major malformations, aneuploidies, depression, or other psychopathological diseases during pregnancy. The GDM group included all GDM patients who delivered in the study period; controls were selected randomly from the EMRs and matched with the GDM group at a 1:2 ratio based on the gestational age at delivery.

According to the American College of Obstetricians and Gynecologists guidelines, GDM diagnosis was conducted using a two-step strategy, consisting of a 50 g glucose challenge test and, if abnormal, followed by a 100 g oral glucose tolerance test [19]. Mothers diagnosed with GDM were instructed to adjust their diet and exercise. Eight women whose glucose was not controlled despite adequate exercise and diet were treated with insulin. Insulin is was considered in for women with fasting glucose levels persisting above 95 mg/dL or those with 2 h postprandial levels > 120 mg/dL. Maternal pre-pregnancy body mass index (BMI) was calculated using the height and weight obtained from the EMR. Gestational weight gain was calculated using maternal body weight at delivery and gestational body weight. Based on the Institute of Medicine/National Research Council 2009 recommendations, we categorized gestational weight gain as low, normal, or high using maternal BMI and gestational weight gain. The recommended weight gain for underweight (BMI < 18.5 kg/m^2^), normal weight (BMI 18.5–24.9 kg/m^2^), overweight (25–29.9 kg/m^2^), and obese (BMI ≥ 30 kg/m^2^) mothers was 12.5–18 kg, 11.5–16 kg, 7–11.5 kg, and 5–9 kg, respectively. Gestational weight gain below, within, and exceeding the recommended range were categorized as “low”, “normal”, and “high”, respectively [20].

The EMR was searched for information about patients and neonates, including demographic, anthropometric, and clinical data. In addition, information on delivery and placental characteristics was compiled. Using Fenton growth curves [21], anthropometric percentiles were calculated for each newborn, standardizing gestational age and gender. Infants with birth weight below the 10th percentile were categorized as small for gestational age (SGA) and those above the 90th percentile as large for gestational age (LGA).

### 2.2. Placental Biopsy

Following placenta delivery, tissue samples for DNA and RNA extraction were collected from the fetal side of the placenta. After removal of the decidua, 0.5 cm^3^ of placental tissue was excised from six random positions in each placenta to avoid inflammation, hemorrhage, infarction, calcification, or fibrin deposition. The tissue samples were stored at −80 °C until further analysis.

### 2.3. Genomic DNA Extraction and Methylation Analysis

Using the GeneAll Exgene Tissue plus SV mini kit (GeneAll Biotechnology, Seoul, Korea), genomic DNA was extracted from 20 µg tissue and evaluated on an Epoch microplate spectrophotometer (Biotek, Winooski, VT, USA). As previously described, we analyzed DNA methylation in *HTR2A* and *SLC6A4* promoter regions [17,22,23]. Ten specific CpG sites were selected from the promoter regions of *SLC6A4* and *HTR2A* (Tables S1 and S2). Pyrosequencing Assay Design Software v2.0 (Qiagen, Hilden, Germany) was used to design PCR amplification and sequencing primers. Table S3 lists the location of the analyzed promoter region and primers relative to *SLC6A4* and *HTR2A*. DNA samples were modified by sodium bisulfite using the EpiTect Fast DNA Bisulfite Kit (Qiagen, #59826). Bisulfite-treated DNAs were amplified using a Pyromark PCR kit (Qiagen, #978703) according to the following program: denaturation at 95 °C for 15 min, followed by 45 cycles at 94 °C, 56 °C, and 72 °C, each for 30 s, and a final extension cycle at 72 °C for 10 min. The amplified PCR products were sequenced using the PyroMark Q48 Autoprep software (Qiagen). The methylation percentage at each CpG site was calculated as the ratio of the peak height of the cytosine signal to the sum of the peak heights of the cytosine and thymidine signals. The methylation percentage of each CpG site was computed using PyroMark Q48 Autoprep 2.4.2. software (Qiagen).

### 2.4. Total RNA Extraction and Gene Expression Analysis

Total human placental RNA was extracted using the RNeasy Plus Mini Kit (Qiagen, Manchester, UK), following the manufacturer’s instructions. RNA purity was determined using a BioPhotometer D30 (Eppendorf, Hamburg, Germany) and 100 ng of purified RNA was reverse-transcribed into first-strand complementary DNA using CellScript cDNA Synthesis Master Mix (CellSafe, Suwon, Korea), which includes a genomic DNA elimination step. RNA expression levels were determined using real-time quantitative PCR on each placental sample. The samples were analyzed using TaqMan gene expression assays (Table S4) (Applied Biosystems, Foster City, CA, USA) on a LightCycler 480 PCR system (Roche, Mannheim, Germany). All assays with comparable amplification efficiencies used a delta cycle threshold for relative quantification. All reactions contained 10 ng of complementary DNA and were run in triplicate using 10 μL of TaqMan probe Master Mix (Roche, Mannheim, Germany). Glyceraldehyde 3-phosphate (GAPDH) served as the housekeeping gene. Results were analyzed using the LightCycler 480 instrument software 1.2 (Roche, Mannheim, Germany) and calculated according to the 2^−ΔΔCT^ method [24].

### 2.5. Statistical Analysis

All statistical analyses were conducted using SPSS version 20.0 (IBM Corp., Armonk, NY, USA) and GraphPad Prism 8.0 (GraphPad Software Inc., La Jolla, CA, USA). Chi-square analysis or Fisher’s exact test was used for categorical data, and Student’s t-test or the Mann–Whitney U test was used for continuous data. Pearson’s correlation analysis was used to examine the association between placental DNA methylation and mRNA expression. Multivariate linear regression with the enter method was used to analyze the correlation of placental DNA methylation levels of SLC6A4 and HTR2A with selected clinical variables. Models were adjusted for maternal age, pre-gestational BMI, gestational age, and newborn sex. For all tests, the significance level was defined as a *p*-value < 0.05.

## 3. Results

### 3.1. Study Participants

Maternal, neonatal, and placental characteristics of the participants were analyzed by comparing the GDM and control groups (Table 1). Women with GDM were older (35.77 ± 2.89 vs. 33.68 ± 4.06 years, *p* = 0.014) and had a higher BMI (26.84 ± 4.52 vs. 22.07 ± 4.04 kg/m*2*, *p* < 0.001) than women in the control group. Gestational weight gain and total cholesterol levels were similar between the two groups. Among neonatal characteristics, the newborn birthweight percentile of the GDM group was higher than that of the control group (54.63 ± 28.53 vs. 40.18 ± 26.28%, *p* = 0.019). The other neonatal characteristics were comparable between the GDM and control groups. In addition, there were no significant differences in placental characteristics between the GDM and control groups.

### 3.2. Methylation Levels of SLC6A4 and HTR2A in Placental Tissues

**Figure 1:**
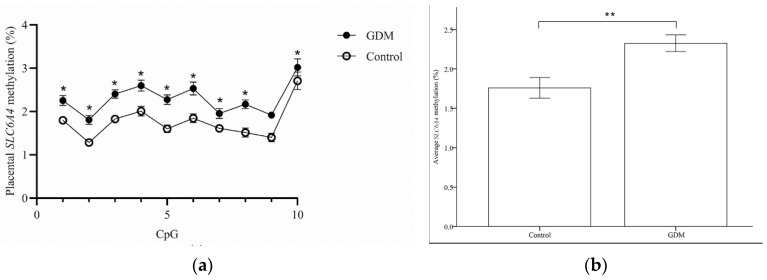
DNA methylation in the promoter region of *SLC6A4*. (**a**) The CpG site methylation patterns on the fetal side of placental biopsies from the GDM and control groups. (**b**) The mean methylation level in the *SLC6A4* promoter region from the third-trimester placenta. * *p* < 0.05; ** *p* < 0.001. GDM: gestational diabetes mellitus.

**Figure 2:**
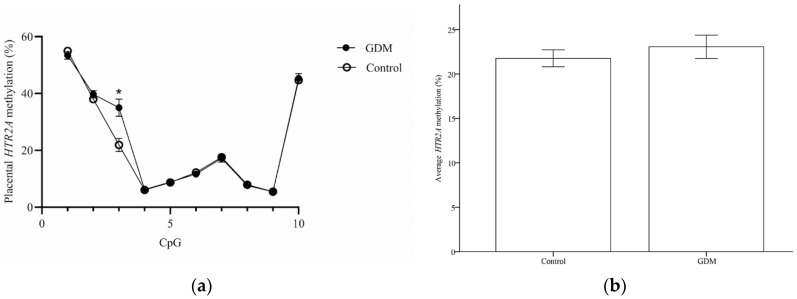
DNA methylation in the promoter region of *HTR2A*. (**a**) The CpG site methylation patterns on the fetal side of placental biopsies from the GDM and control groups. (**b**) The mean methylation level in the *HTR2A* promoter region from the third-trimester placenta. * *p* < 0.05, GDM: gestational diabetes mellitus.

Placental tissues were analyzed for *SLC6A4* and *HTR2A* DNA methylation levels. The *SLC6A4* DNA methylation levels at 9 of 10 CpG sites were significantly higher in the GDM group than in the control group (Figure 1a), and the mean *SLC6A4* DNA methylation was also higher in the GDM group (2.29 ± 0.06 vs. 1.16 ± 0.07%; *p* < 0.001; Figure 1b). However, there were no significant differences in the mean *HTR2A* methylation in the placenta between women with or without GDM (23.07 ± 0.64 vs. 21.76 ± 0.47; *p* = 0.111; Figure 2b). Among individual CpG analyses, only the H3 level was significantly elevated in the GDM group (35.04 ± 3.03 vs. 21.93 ± 2.28%; *p* = 0.001; Figure 2a).

### 3.3. mRNA Expression of the Serotonin System

**Figure 3:**
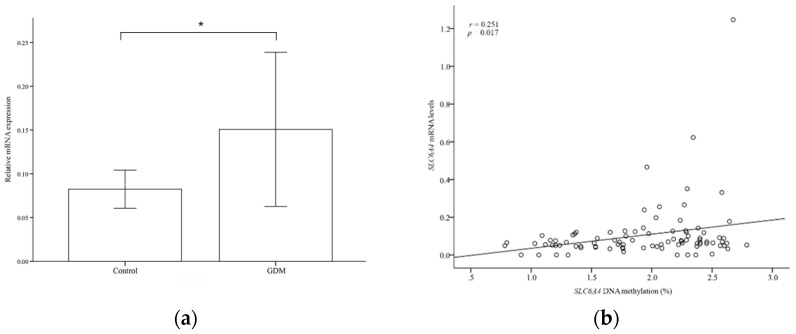
*SLC6A4* mRNA expression in placental tissue. (**a**) Analysis of relative *SLC6A4* messenger ribonucleic acid (mRNA) levels in GDM and control placentas. (**b**) Placental *SLC6A4* methylation levels are correlated with placental *SLC6A4* mRNA levels. * *p* < 0.05, GDM: gestational diabetes mellitus.

We assessed the placental mRNA expression of *SLC6A4* and *HTR2A* using quantitative real-time PCR. As shown in Figure 3a, GDM patients had significantly higher *SLC6A4* mRNA expression than controls (0.15 ± 0.43 vs. 0.08 ± 0.10%, *p* = 0.048). However, *HTR2A* mRNA expression was nearly absent in both the GDM and control groups. We quantified the association between *SLC6A4* mRNA levels and DNA methylation to determine whether the *SLC6A4* methylation markers associated with GDM have functional significance. A significant positive correlation was found between *SLC6A4* methylation and placental *SLC6A4* mRNA level (r = 0.251, *p* = 0.017; Figure 3b).

### 3.4. Correlations between Placental HTR2A and SLC6A4 Methylation Levels and Clinical Factors

We investigated whether placental DNA methylation levels of *SLC6A4* and *HTR2A* were correlated with maternal and newborn clinical factors. By adjusting for maternal age, BMI, gestational age, and newborn sex as confounding factors, correlations were investigated using multivariate linear regression (Table 2). *SLC6A4* methylation showed a significant positive correlation with fasting plasma glucose levels (β = 0.007, *p* = 0.036) and birth weight percentiles (β = 0.008, *p* = 0.002). The mean *SLC6A4* methylation level was negatively correlated with the neonatal HC percentile (β = −0.006, *p* = 0.017). However, *HTR2A* DNA methylation levels were not correlated with maternal glucose levels or with clinical factors of the newborn.

## 4. Discussion

To our knowledge, this study is the first to analyze the potential effect of maternal GDM on the neonatal outcome by the placental serotonin epigenotype. In particular, we investigated the relationship between GDM and placental DNA methylation in the serotonin system. Placentas in the GDM group showed significantly increased *SLC6A4* methylation compared to those in the control group. We found a positive correlation between *SLC6A4* methylation and maternal plasma glucose levels and neonatal birth weight percentile. This correlation remained statistically significant after adjusting for potential confounders, including pre-pregnancy BMI, maternal age, and neonatal sex. This suggests that intrauterine hyperglycemia affects *SLC6A4* promoter hypermethylation and offspring development. In addition, *SLC6A4* methylation was negatively correlated with neonatal HC percentile, which may be linked to neurodevelopmental impairment.

The mean placental *SLC6A4* methylation level was higher in GDM patients than in controls and was positively correlated with *SLC6A4* mRNA levels. However, previous studies have reported contradictory results. In a small cohort study, placental *SLC6A4* mRNA expression was lower in GDM pregnancies [18]. However, another study reported decreased *SLC6A4* methylation and increased *SLC6A4* mRNA expression in the placentas of GDM pregnancies compared to those in controls [22]. In addition, we found a positive correlation between promoter methylation levels and gene expression, despite CpG methylation inhibiting gene transcription [25]. The serotonergic system in the placenta may be influenced by additional factors, accounting for these contradictory findings. Participants in these studies showed varying degrees of maternal obesity, which might interfere with the serotonin system by regulating appetite and energy balance [26]. In addition, the maternal and neonatal serotonin systems may influence the placental serotonin system. In the early stages of fetal development, the placenta synthesizes serotonin using maternal tryptophan, the precursor of serotonin, and in later stages until delivery, the fetus can synthesize serotonin by itself [13]. Another explanation for the discordance could be differences in the study population. While previous studies only focused on full-term pregnancy after 37 weeks, in the present study, we included women with preterm births. There were also racial differences. Further study should investigate the different effects of race on placental serotonin system.

Interestingly, placental *SLC6A4* DNA methylation was negatively correlated with the neonatal HC percentile. No prior study has evaluated the relationship between the placental serotonin system and infant HC. However, our findings are consistent with a previous study showing that higher stress in very-preterm infants with a greater increase in *SLC6A4* methylation is associated with reduced brain volume on MRI performed at term-equivalent age [27]. Both increased and decreased placental serotonergic activity can affect fetal brain development [28]. ASD has been linked to deficiencies in placental serotonin [29,30], and hyperserotonemia during fetal development may trigger a negative feedback loop that suppresses serotonin [28]. Moreover, *SLC6A4* downregulation in mouse brains alters cortical organization and results in an ASD-associated phenotype. Thus, the present findings suggest that the stress of maternal hyperglycemia in the fetus might be associated with reduced brain volume in infants via epigenetic regulation of the serotonin transporter gene.

Placental *SLC6A4* DNA methylation was positively correlated with neonatal birth weight percentile. Increased serum serotonin levels have been associated with obesity in mice [31]. In previous studies, lower *SLC6A4* CpG methylation in the umbilical cord was associated with the infant to adult adiposity [32]. However, in an adult twin study, higher *SLC6A4* promoter methylation in peripheral blood leukocytes was associated with obesity [33]. This discrepancy may be attributed to the different CpG site locations and the analyzed tissues.

Unlike the DNA methylation levels of the serotonin transporter, placental *HTR2A* mRNA expression and DNA methylation were not altered in the GDM group compared to those in the controls. This finding suggests that placental receptor subtypes other than *HTR2A* may be involved in the association between maternal GDM and neonatal outcomes. Further studies are required to elucidate the possible mechanisms underlying GDM and serotonin receptors.

One of the strengths of our study is the well-characterized gestational age-matched cohort. Moreover, the ethnic homogeneity of our population helped avoid discrepancies and confounding effects. The present study is the first to identify unique associations between prenatal GDM and the neonatal brain. Our study is also the first to investigate placental serotonin signaling pathways as moderators of the association between prenatal GDM exposure and neonatal development. However, this study has some limitations. First, the sample size is small. Given the complexity of the findings and the small sample sizes for sex-stratified analyses, replication of these findings in an independent and larger cohort is needed. Second, the present study did not investigate generic serotonin variants. Third, some women were treated with insulin, whereas others had diabetes under control with diet and exercise alone. There was no difference in glucose levels between the two groups; we, therefore, thought that there was little effect on this experiment. Fourth, several demographic characteristics, such as social, economic, and emotional factors, may have influenced our results. Future prospective and longitudinal research is warranted to elucidate the intricate interplay between maternal GDM, epigenetic variations, and fetal development during pregnancy in contributing to the long-term outcomes of offspring.

In summary, the expression of the serotonin transporter is dysregulated in the placenta of GDM-affected pregnant women relative to that in the control pregnant women. These findings improve our understanding of the placental adaptive response mechanisms to maternal hyperglycemia, which increases the risk of adverse long-term outcomes for the developing fetus.

## Figures and Tables

**Table 1:** Baseline characteristics of the study population.

	GDM (N = 30)	Control (N = 60)	*p*-Value
Maternal characteristics			
Maternal age, years	35.77 ± 2.89	33.68 ± 4.06	0.014 *
Nulliparous, %	15 (50)	40 (66)	0.262
Prepregnancy BMI, kg/m^2^	26.84 ± 4.52	22.07 ± 4.04	<0.001 *
Gestation at birth, weeks	36.91 ± 2.46	37.13 ± 2.31	0.683
Delivery < 37 weeks, %	10 (33.3)	18 (30)	0.747
Gestational weight gain	10.97 ± 7.58	13.75 ± 5.65	0.054
% low category	13 (43.3)	19 (31.7)	0.548
% normal category	9 (30.0)	21 (35.0)	
% high category	8 (28.6)	20 (33.3)	
Fasting glucose, mg/dL	96.47 ± 18.59	86.62 ± 15.62	0.01 *
50 g OGTT	186.06 ± 30.03	122.70 ± 21.61	< 0.001 *
Total cholesterol	256.07 ± 41.85	267.26 ± 50.75	0.431
Neonate and placenta characteristics			
Birth weight, kg	3.03 ± 0.76	2.86 ± 0.72	0.3
Birthweight percentile	54.63 ± 28.53	40.18 ± 26.28	0.019 *
SGA, %	1 (3.3)	6 (10.0)	0.205
LGA, %	5 (16.7)	4 (6.7)	
Apgar < 7 at 5 min, %	1 (3.3)	2 (3.3)	1
Infant gender (% male)	13 (43.3)	33 (55.0)	0.297
Fasting glucose	79.14 ± 55.11	64.25 ± 28.51	0.21
TSH	3.63 ± 2.17	3.77 ± 2.14	0.769
17alpha-OHP	1.83 ± 1.15	2.04 ± 1.20	0.433
Placenta weight, kg	0.70 ± 0.21	0.64 ± 0.20	0.192
Placental weight < 10th percentile	2 (6.7)	9 (15.0)	0.255

The values are expressed as mean ± standard deviation or number (%). * *p* < 0.05. GDM: gestational diabetes mellitus; BMI: body mass index; OGTT: oral glucose tolerance test; SGA: small for gestational age; LGA: large for gestational age; TSH: thyroid stimulating hormone; OHP: hydroxyprogesterone.

**Table 2:** Multivariate linear models analyzing the correlation of *SLC6A4* methylation and *HTR2A* methylation with selected clinical variables.

	β	*p*	R^2^
Mean methylation levels of *SLC6A4* (%)			
Fasting plasma glucose	0.007	0.036 *	0.196
Birthweight percentile	0.008	0.002 *	0.197
HC percentile	−0.006	0.017 *	0.226
Mean methylation levels of *HTR2A* (%)			
Fasting plasma glucose	0.037	0.132	0.061
Birthweight percentile	0.015	0.441	0.006
HC percentile	−0.018	0.350	0.072

Models were adjusted for maternal age, pre-gestational BMI, and neonatal sex. * *p* < 0.05. HC: head circumference.

## Data Availability

The data presented in this study are available on request from the corresponding author.

## Supplementary Materials

The following supporting information can be downloaded at: https://www.mdpi.com/article/10.3390/life12111869/s1, Table S1: CpG site position on chromosome of the *SLC6A4* gene, Table S2. CpG sites position on chromosome of the *HTR2A* gene, Table S3: Primer sets for quantitation of site-specific *HTR2A*, *SLC6A4* CpG methylation by pyrosequencing, Table S4: Gene expression assays used for Real-time PCR for human placental tissue samples.
