# Supplementary material for: Maternal Gestational Diabetes Influences DNA Methylation in the Serotonin System in the Human Placenta
Source: Life (Basel). 2022 Nov 13;12(11):1869. doi: 10.3390/life12111869 (PMC9695704; doi:10.3390/life12111869)
Supplement: Supplementary file 1 [file life-12-01869-s001.zip › life-1992935-supplementary.pdf]

Table S1. CpG site position on chromosome of the *SLC6A4* gene.

CpG progressive number	Dinucleotide position on chromosome 17	Distance from the TSS [a]
1	Chr 17 : 28563253-28563254	-235
2	Chr 17 : 28563237-28563238	-219
3	Chr 17 : 28563226-28563227	-208
4	Chr 17 : 28563143-28563144	-125
5	Chr 17 : 28563138-28563139	-120
6	Chr 17 : 28563120-28563121	-102
7	Chr 17 : 28563109-28563110	-91
8	Chr 17 : 28563111-28563112	-93
9	Chr 17 : 28563101-28563102	-83
10	Chr 17 : 28563090-28563091	-72

[a] TSS : Transcription start site.

Table S2. CpG sites position on chromosome of the *HTR2A* gene.

CpG progressive number	Dinucleotide position on chromosome 13	Distance from the TSS [a]
1	Chr13 : 47471563-47471564	-1392
2	Chr13 : 47471479-47471480	-1306
3	Chr13 : 47471460-47471461	-1289
4	Chr13 : 47471198-47471199	-1027
5	Chr13 : 47471169-47471170	-998
6	Chr13 : 47471058-47471059	-887
7	Chr13 : 47471053-47471054	-882
8	Chr13 : 47471029-47471030	-858
9	Chr13 : 47471024-47471025	-853
10	Chr13 : 47470694-47470695	-521

[a] TSS : Transcription start site.

Table S3. Primer sets for quantitation of site-specific *HTR2A, SLC6A4* CpG methylation by pyrosequencing.

Primer	Region	Amplification primers[a]	Sequencing primers
HTR2A	1	F: TGGTTATATAGGTTTAGGGTGGTTAG<br>R: ACCAACTTATTTCCTACCACATAT	TTAGGGTGGTTAGGT
	2	F: TAGGTTGAAGGGTGAAGAGAGAAT<br>R: CACCCTAAACCTATATAACCAATATCA	ATAAGGTTAGAAAATAGTATGTT
	3	F: AAGAATTGTATGGGAAAGTAGGA<br>R: ATTTAATCCCCCCTCCTAACTATTACTACC	TGTATTAAGGGATTTTTGG
	4	F: GAGGGGTTAGTGAATGATTTTTAAATGTGT<br>R: ACTCCCCCACTACTAAAATCCTATTAACTT	ATTTTTAAATGTGTGTTTGTTG
	5	F: ATGTGTGGATTTTGAAGATAAATGTAAG<br>R: ACAACTTTCCTCCCTAAAAATTCT	GGATTTTGAAGATAAATGTAAGT
SLC6A4	1	F: ATTAGATAAGGGTTTTTAAGTTGAGTT<br>R: TCCCCAAAACTACTCTCTCTTTCTTCC	GGGTTTTTAAGTTGAGTTTATA
	2	F: GGGAGGTGTTAGAGGTTAAGAGAA<br>R: ATCCTAACTTTCCTACTCTTTAACT	GGTGTTAGAGGTTAAGAGAAA

[a] F forward primer, R reverse primer (5'biotin).

Table S4. Gene expression assays used for Real-time PCR for human placental tissue samples.

Gene	Abbreviation	Reference Sequence	Assay number
5-hydroxytryptamine receptor 2A	HTR2A	NM_000621	Hs01033524_m1
solute carrier family 6 member 4	SLC6A4	NM_000619	Hs00984349_m1
Glyceraldehyde 3-phosphate	GAPDH	NM_002046	Hs99999905_m1
